# Catheter-Directed Thrombolysis for Acute Superior Mesenteric Artery Occlusion: A Case Report

**DOI:** 10.7759/cureus.107921

**Published:** 2026-04-28

**Authors:** Manogna Nuthi, Raydiene Doorgen, Sean S Wale, Suemoy Wallace

**Affiliations:** 1 Department of Surgery, Midwestern University Arizona College of Osteopathic Medicine, Glendale, USA; 2 Department of Surgery, HonorHealth, Scottsdale, USA; 3 Department of Surgery, Abrazo Health, Glendale, USA

**Keywords:** acute mesenteric ischemia (ami), catheter-directed thrombolysis, minimally invasive surgery, occlusive mesenteric ischemia, superior mesenteric artery (sma), thromboembolism

## Abstract

Acute mesenteric ischemia (AMI) is the sudden onset of small intestinal hypoperfusion, commonly due to thromboembolic obstruction of mesenteric vessels. Due to nonspecific symptoms at presentation, diagnosis can often be delayed until the condition rapidly progresses. Additionally, these factors likely contribute to AMI’s high mortality rate, highlighting the importance of early diagnosis and intervention. Traditionally, AMI is managed with open abdominal surgery (e.g., embolectomy, bowel resection). However, alternative treatment methods have evolved, including endovascular techniques such as thrombolysis, which offer targeted therapy while preserving bowel integrity. The following case presents the management of a patient with mild mesenteric ischemia treated with the lesser-utilized catheter-directed thrombolysis.

## Introduction

Acute mesenteric ischemia (AMI) is a life-threatening condition that carries a high short-term mortality rate of 60-90% [1,2]. AMI has been associated with a poorer prognosis due to the presentation of non-specific symptoms and rapidly progressing necrosis of the small and, rarely, large intestines that leads to sepsis and death [1]. Hence, accurate diagnosis and timely intervention remain a significant challenge. The prevalence of this condition remains one in 1000, with higher incidence in women, the elderly, and those with multiple comorbidities [1,3].

The pathophysiology of AMI involves sudden small intestinal hypoperfusion with accelerated development. There are two etiologies of mesenteric ischemia: occlusive and non-occlusive. Occlusive mesenteric ischemia (OMI) can be further categorized into arterial and venous types, with arterial being the most common at approximately 60%, while venous accounts for around 10% [1-3]. Non-occlusive mesenteric ischemia (NOMI) represents the remaining 30% of cases [2,3]. Moreover, the superior mesenteric artery (SMA) is most frequently affected due to its narrow anatomic angle [4-6]. Approximately 15% of emboli remain at the origin of the SMA, while the remaining tend to lodge 3-10 cm more distally [6]. Further classification of arterial OMI distinguishes between embolic and thrombotic obstructions, with embolic obstructions more commonly identified and thrombotic obstructions associated with the worst prognosis due to frequent proximal lodging in the SMA [4-6]. The most likely source of embolism in OMI is the heart, which includes thrombi from the atrial appendage secondary to atrial fibrillation, ventricular mural thrombi secondary to hypokinesis after myocardial infarction, or from valvular origins [1,2,4,5,7].

Furthermore, the diagnosis of AMI relies on the current gold standard, computed tomographic angiography (CTA), for both acute and chronic mesenteric ischemia, with catheter-based angiography as a second-line modality due to its invasive nature and limited accessibility [6]. Despite non-specific findings of bowel wall thickening, hyper- or hypo-enhancement, and filling defects in the major mesenteric arteries, identification of these signs on imaging should raise suspicion for AMI in conjunction with the clinical presentation [6]. Initial symptomology can include abdominal pain that does not correlate with exam, nausea, diarrhea, bloody stool, and distension [1,7]. As the condition rapidly progresses, it can involve severe tenderness to palpation, which correlates with the development of bowel necrosis [1]. Lastly, laboratory findings remain lower on the diagnostic ladder, as they are non-specific to mesenteric ischemia, but they can bolster clinical suspicion for AMI as it worsens. Leukocytosis, elevated lactate, and elevated D-dimer can support and guide further workup with imaging for ischemia and prompt initiation of treatment [1,2,5,7].

Traditional management for AMI involves open abdominal surgery for prompt revascularization, removal of necrotic bowel, and preservation of viable tissue [5]. However, recent developments have identified endovascular approaches as a viable, and possibly better, alternative to open surgery when available; yet, limited access to specialists raises concerns about delays in revascularization, leading to progressed necrosis that serves as a contraindication to this intervention [5,8]. Here, we present a case that uses catheter-directed thrombolysis to treat a patient with AMI, enabling a minimally invasive approach and early intervention that prevents acute decompensation.

## Case presentation

An 85-year-old female with a past medical history of hypertension, left breast carcinoma status post mastectomy (on the chemotherapeutic agent, Letrozole), and hypothyroidism presented to the emergency department complaining of acute onset, sharp epigastric pain. The patient denied any prior history of venous or arterial thromboembolism or any family history of hypercoagulable disorders. The patient stated that her pain started the night prior to presentation, after a meal, and was radiating to the back. She also reported associated dry heaves and regular bowel movements. She denied any previous episodes of similar symptoms.

**Figure 1:**
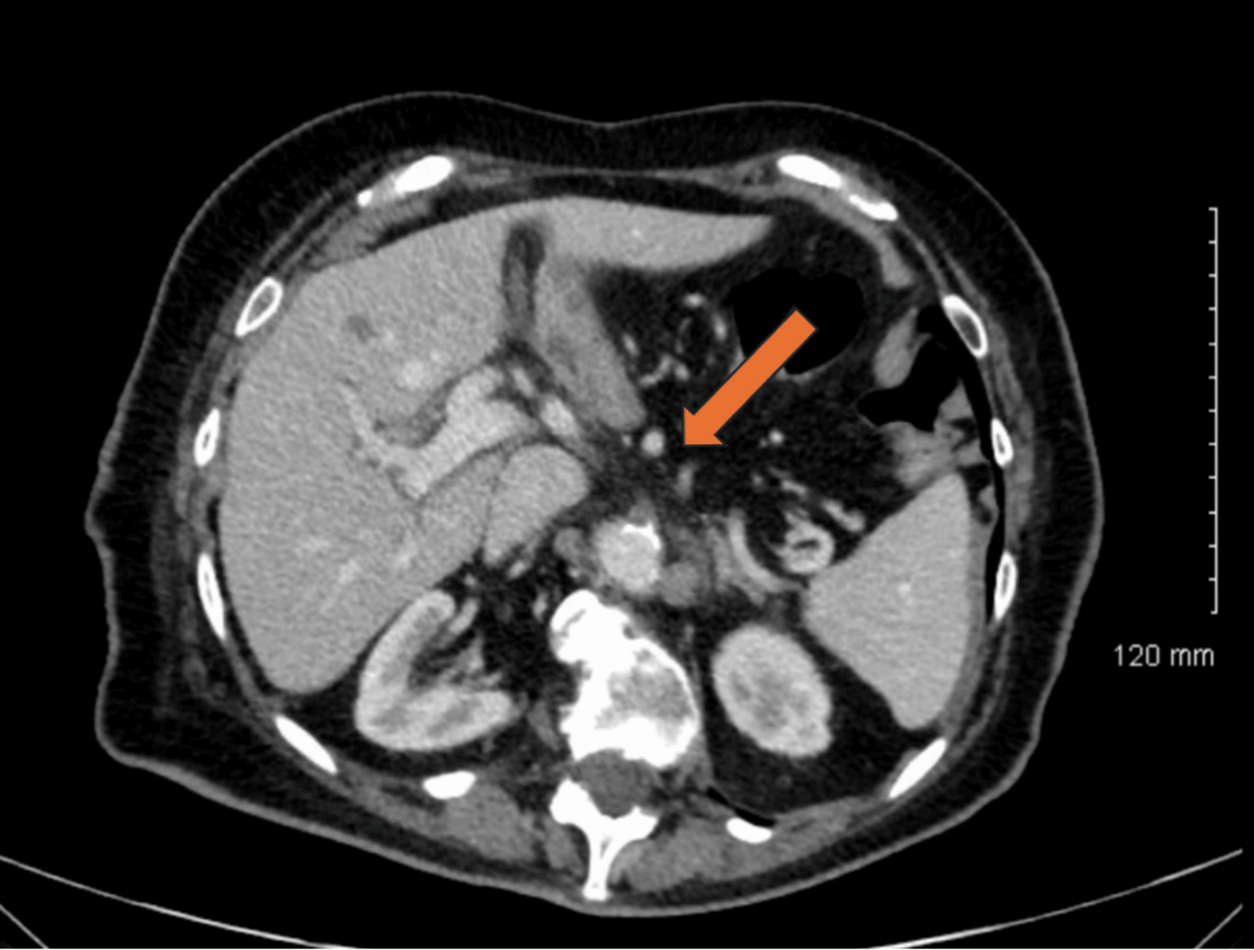
Axial section of a CT of the abdomen showing focal lack of enhancement in the proximal superior mesenteric artery.

**Figure 2:**
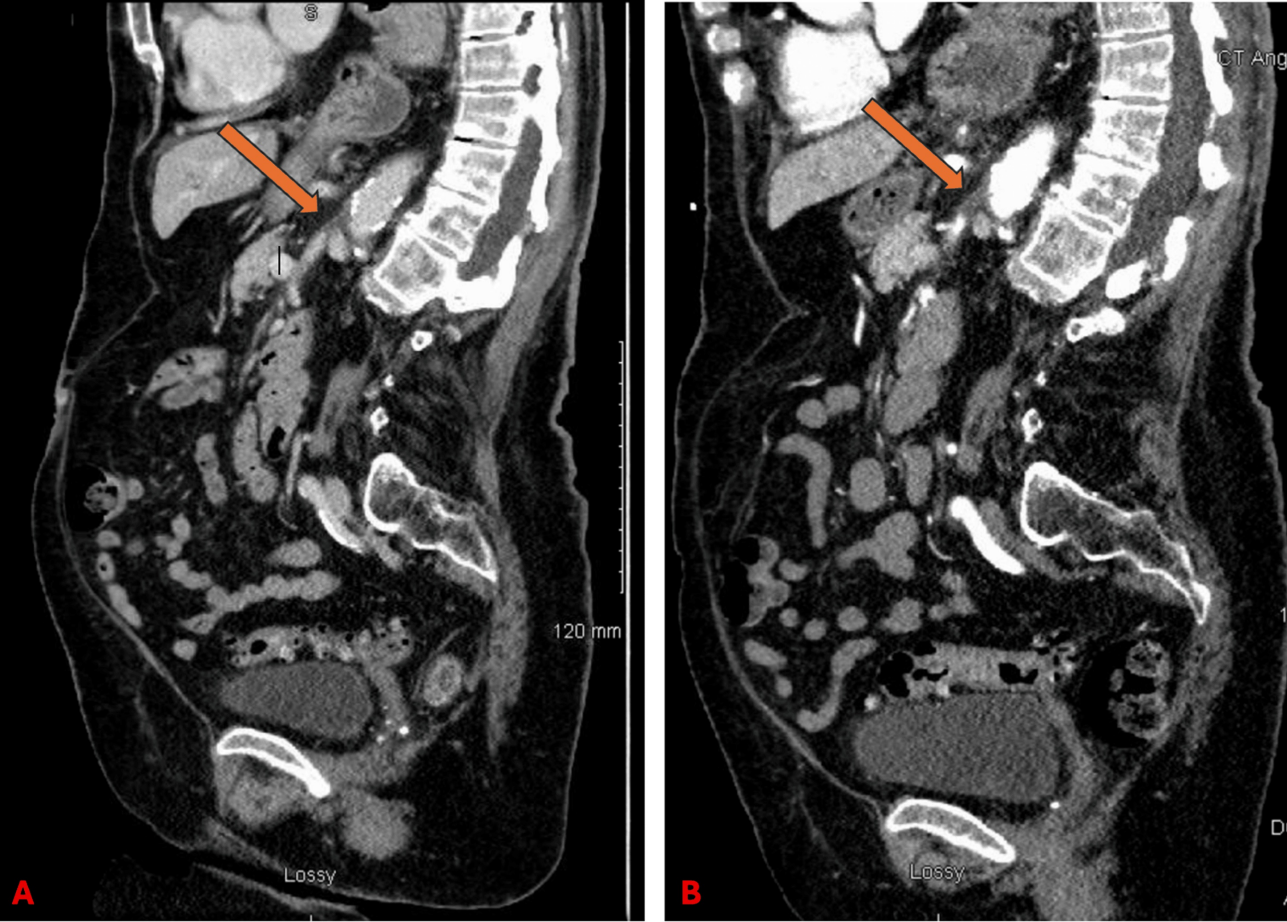
Sagittal section of a CT of the abdomen indicating retrograde filling (A) and the superior mesenteric artery (B).

On presentation, her abdomen was benign on physical examination. Vital signs were stable, white blood cell count was slightly elevated at 11.5 cells/µL, and serum lactate was 2.1 mg/dL. A computed tomography (CT) of the abdomen and pelvis demonstrated focal lack of enhancement of the proximal SMA (Figure 1), with distal flow indicating retrograde filling (Figures 2A-2B). Findings were concerning for an SMA occlusion. Interventional radiology was consulted for prompt intervention. A continuous heparin infusion and broad-spectrum antibiotic coverage with piperacillin/tazobactam were initiated. Within two hours, the patient then underwent fluoroscopically guided placement of a 5-French catheter at the origin of the SMA via percutaneous access in the right common femoral artery. Tissue plasminogen activator (tPA) was continuously infused via the catheter at a rate of 1mg per hour for 24 hours, and a continuous heparin infusion was started utilizing the Anti-Xa titration protocol. The patient was closely monitored in the intensive care unit with serial abdominal examinations assessing for signs of peritonitis and/or abdominal compartment syndrome.

The patient was taken back for follow-up angiography 24 hours later. Repeat angiography revealed evidence of a persistent thrombus at the origin of the SMA. Patent branches of the SMA receiving collateral inflow from the inferior mesenteric artery (IMA) via the marginal artery of Drummond and a patent arc of Buhler were also evident. During this time, the patient showed no progression of her symptoms, complaining only of mild, vague abdominal pain. Abdominal examination remained normal with no distension, tenderness, guarding, or rigidity. She continued to have regular non-bloody bowel movements. With evidence of adequate collateral inflow and minimal concern for bowel compromise, further intervention was deemed unnecessary. The following day, the patient was initiated on a loading dose of Eliquis at 10 mg twice per day for seven days, then transitioned to 5 mg twice daily for six months.

Throughout her hospital course, she underwent a workup for the underlying etiology of arterial thromboembolism. An echocardiogram performed on hospital day two ruled out an intracardiac origin of embolism. Coagulation studies, including prothrombin time (PT), international normalized ratio (INR), and partial thromboplastin time (PTT), were all within normal limits. Additional workup for protein C/S deficiency and Factor V Leiden was negative. She was recommended to follow up with her established hematology oncologist for additional workup of other underlying causes of hypercoagulability, such as cancer recurrence. Follow-up CT imaging two months after discharge demonstrated persistent proximal SMA occlusion with collateral filling, a widely patent IMA, and no evidence of bowel ischemia. The patient remains on Eliquis 5 mg daily.

## Discussion

AMI is a rare yet life-threatening condition that necessitates timely and accurate diagnosis to optimize patient outcomes. This case highlights the potential of catheter-directed thrombolysis as an effective treatment for thromboembolic occlusion of the SMA. The patient’s clinical presentation, characterized by vague abdominal pain and the absence of severe ischemic symptoms, underscores the diagnostic challenges associated with AMI. Early intervention and close multidisciplinary collaboration led to a favorable outcome, underscoring the importance of individualized treatment strategies.

The traditional management of AMI often involves open surgical interventions, including embolectomy and bowel resection. However, advancements in endovascular techniques have enabled less invasive approaches with outcomes comparable to or better than those achieved with traditional approaches. Additional endovascular techniques include percutaneous mechanical and aspiration thrombectomy, which involve fragmenting of thrombus and thrombus aspiration, respectively, through catheters [9]. As seen in this case, catheter-directed thrombolysis offers the advantage of directly targeting the thrombus while preserving bowel integrity and reducing the risks associated with open surgery. Studies, such as those by Malhotra et al. [10] and Schoots et al. [11], have highlighted the efficacy of thrombolysis in selected patients, particularly when initiated promptly after symptom onset. These studies support this patient’s outcomes, demonstrating successful reperfusion without surgical intervention. However, the persistence of a thrombus in the SMA, despite adequate collateral flow, raises questions about the long-term implications and the need for ongoing anticoagulation therapy to mitigate future risks. Malhotra et al. noted only a residual clot in their patient, reflecting a greater reduction in clot size than in our patient [10]. However, this could be due to differences in the thrombolytic agent, as they used Retavase in a pulse-spray method over 30 minutes rather than tPA with continuous infusion, as in our case [10]. Moreover, the role of collateral circulation, as evidenced by the patent IMA and the marginal artery of Drummond in this case, cannot be understated. Adequate collateral flow likely contributed to the absence of bowel ischemia despite the proximal SMA occlusion. This observation highlights the need to account for anatomical variations and the adequacy of collateral perfusion when evaluating treatment options.

While catheter-directed thrombolysis is a promising option, it is not without limitations. There is a limitation on access to and availability of interventional radiologists who can perform this technique. In our case, treatment initiation within a reasonable time frame without delayed revascularization was possible. Additionally, potential complications of catheter-directed thrombolysis include bleeding, reperfusion injury, and incomplete thrombus resolution, as observed in this case [10]. Future studies should focus on refining patient selection criteria, optimizing dosing protocols for thrombolytic agents, and exploring adjunctive therapies to enhance treatment efficacy. Lastly, long-term follow-up studies are necessary to assess the durability of outcomes and the risk of recurrent thromboembolism with this approach.

## Conclusions

Catheter-directed thrombolysis represents a promising, minimally invasive treatment option for acute SMA occlusion in carefully selected patients without clear signs of bowel ischemia or necrosis. This case highlights the importance of individualized management, early diagnosis, vigilant monitoring, and close interdisciplinary collaboration in achieving favorable outcomes while avoiding open surgery. The successful preservation of bowel integrity underscores the potential of thrombolysis to reduce morbidity in patients who may not tolerate more invasive procedures. As management of AMI continues to evolve, further research is needed to better define patient selection criteria, optimize thrombolytic protocols, and evaluate long-term outcomes. This case contributes meaningful insights to an emerging paradigm shift toward less invasive interventions for thromboembolic AMI.
